# Retinal disease classification from OCT images using EfficientNetB3 with neutrosophic similarity score enhancement

**DOI:** 10.1002/acm2.70573

**Published:** 2026-05-14

**Authors:** Devi M, G. Hannah Grace

**Affiliations:** ^1^ Department of Mathematics School of Advanced Sciences Vellore Institute of Technology Chennai Chennai Tamil Nadu India

**Keywords:** Deep learning, EfficientNetB3, Neutrosophic Sets, Neutrosophic Similarity Score, Optical Coherence Tomography, Retinal Disease Classification

## Abstract

**Purpose:**

Automated classification of retinal diseases from optical coherence tomography (OCT) images plays a significant role in supporting early diagnosis and clinical decision‐making. In this study, a deep learning (DL) framework is developed by integrating a neutrosophic similarity score (NSS) with the EfficientNetB3 to improve image representation and classification accuracy.

**Methods:**

Initially, OCT images are transformed into the neutrosophic domain, where NSS is combined with contrast limited adaptive histogram equalization (CLAHE) and applied to enhance informative structures by reducing noise, ambiguity and indeterminacy. Then the enhanced images are directly utilized for training an EfficientNetB3 model. A two‐phase training strategy is adopted, that consist of initial training with frozen base layers and then followed by fine‐tuning of deeper layers. The performance of the proposed approach is evaluated on a retinal OCT dataset and compared with that of conventional DL models trained on the original images.

**Results:**

Experimental results show that the proposed NSS‐integrated EfficientNetB3 model achieved an overall accuracy of 95.25%, along with precision, recall, and F1‐score of 95.33%, 95.25% and 95.26%, respectively. The model demonstrates improved performance when compared to models trained on the original OCT images.

**Conclusions:**

The results indicate that the integration of NSS with DL improves discriminative feature learning and classification performance. The proposed study highlights the effectiveness of neutrosophic‐based enhancement techniques for advancing automated retinal disease classification.

## 1 INTRODUCTION

Early and accurate diagnosis is important for effective treatment of retinal diseases, which remain a leading cause of vision damage and blindness all over worldwide. The OCT imaging technique was introduced by Huang et al.,^1^ and has become a fundamental imaging modality in ophthalmology due to its ability to provide high‐resolution cross‐sectional imaging of retinal structures in a non‐surgical method. Despite its clinical utility, the complex and frequently overlapping characteristics of retinal diseases make manual interpretation challenging, which creates a need for reliable automated classification systems to enhance diagnostic accuracy and efficiency. Clinically significant retinal conditions are identifiable through OCT which includes Age‐related macular degeneration (AMD), central serous retinopathy (CSR), choroidal neovascularization (CNV), diabetic retinopathy (DR), diabetic macular edema (DME), drusen and macular hole (MH), all of which are associated with structural retinal alterations that may lead to progressive vision loss.^2^, ^3^, ^4^, ^5^, ^6^ In recent years, the automated analysis of OCT images has added important attention as a solution to the limitations of manual diagnosis. The increasing difficulty of retinal diseases and the large volume of clinical imaging data make manual interpretation time‐consuming and vulnerable to inter‐observer variability. The DL models have demonstrated strong potential by automatically learning hierarchical feature representations from large datasets that enable highly accurate predictions.^7^, ^8^ In particular, CNNs have been broadly applied for OCT‐based disease classification and achieving reliable detection of conditions such as AMD, CNV and DME.^9^ Advanced architectures like ResNet, DenseNet and EfficientNet, have further improved the performance by addressing challenges related to network deepness, vanishing gradients and computational efficiency.^10^, ^11^, ^12^

Despite of these advances, OCT analysis still faces challenges due to dissimilarities in image quality. Common factors such as noise, poor contrast and refined structural alterations can hinder reliable feature extraction and consequently reduce classification accuracy. To address these challenges, researchers have investigated on various image enhancement strategies. Among them, neutrosophic theory has developed as an auspicious approach for medical image preprocessing. Unlike the traditional filtering or fuzzy set methods, neutrosophic representation explicitly models truth (T), indeterminacy (I) and falsity (F), which makes it effective in handling uncertainty and noise.^13^ Applications of neutrosophic sets (NS) in medical imaging have demonstrated better performance in tasks like brain tumor detection, breast cancer analysis and retinal layer segmentation.^14^

Enhancing the medical image quality is vital for accurate diagnosis and clinical decision‐making. Preprocessing and enhancement techniques are especially important in OCT imaging, where they help reveal delicate structural details by suppressing noise and reducing uncertainty. Among the existing approaches, neutrosophic approaches have gained attention due to their ability to explicitly represent indeterminacy and ambiguity. Chaira^15^ introduced NS for mammogram image enhancement using the T, I and F components for improving brightness and preserving fine details through divergence score and modified histogram hyperbolization. For denoising, the NS‐based methods transform images into neutrosophic domain and apply γ‐median filtering with neutrosophic entropy, that enables effective noise removal without prior knowledge of noise characteristics.^16^ Similarly, the neutrosophic‐based edge detection practices directional α‐mean operations to suppressing the noise and enhance edge representation.^17^ Although NSS‐based enhancement methods further improves contrast and structural preservation which supports computer‐aided diagnosis.^18^ Several neutrosophic filtering strategies, including bilateral, Wiener, median, Gaussian and rank‐ordered filters, have also demonstrated higher performance over conventional techniques.^19^ More recently, the Type‐2 neutrosophic approach was proposed to further reduce uncertainty and enhance contrast in medical images.^20^ Neutrosophic methods have been applied in segmentation, restoration and image accomplishment by leveraging T, I and F domains with entropy‐based uncertainty reduction. The α‐means clustering enables strong segmentation of both clean and noisy images,^21^ while neutrosophic image completion expands patch matching and hole filling with better ASVS, MSSIM and PSNR performance.^22^ Watershed‐based segmentation in the neutrosophic domain additionally improves structural description.^23^ In addition, linguistic neutrosophic cubic sets (LNCS) enhance the uncertainty modeling in noisy grayscale images by improving visual quality and computational efficiency.^24^ Recent work extends these methods to broader applications, which includes NSS‐based enhancement for improved illumination, contrast and color balance in pathological microscopic images,^25^ and then the hybrid optimization combining with salp swarm algorithm (SSA) and neutrosophic modeling to enhance dark regions in skeletal scintigraphy images.^26^ Similarly, the NSS‐based thresholding methods have also shown strong toughness across clean and noisy conditions.^27^

NS theory has also been integrated with DL for medical image analysis. Amin et al.^28^ proposed a breast tumor classification approach combining texture and morphological features with NSS and achieving 99.1% accuracy by addressing speckle noise and low contrast in ultrasound images. Similarly, Alsattar et al.^29^ developed an active localization‐driven approach using probabilistic neutrosophic hesitant fuzzy sets to evaluate Hybrid Multi Deep Transfer and Machine Learning models for COVID‐19 chest X‐ray classification. In addition, studies on OCT angiography (OCTA) for Behçet uveitis diagnosis have combined AI models with clinical data to improve diagnostic performance and achieve an AUC of 0.908.^30^ Although these studies demonstrate the efficiency of neutrosophic‐DL integration and their applications mainly focus on feature fusion, decision outlines or modality‐specific analyses rather than enhancement‐determined OCT classification. Therefore, a clear gap remains in leveraging NSS‐based image enhancement directly within a DL pipeline for multi‐class OCT retinal disease classification. This study addresses this gap by presenting a framework that enhances OCT images using the NSS method and integrates the enhanced representations with the EfficientNetB3 architecture to improve classification accuracy and overall performance.

Motivated by these understandings, the present work introduces a hybrid approach that combines neutrosophic preprocessing with an EfficientNetB3 model for OCT image analysis. Neutrosophic preprocessing improves image quality by suppressing noise and emphasizing serious retinal features, while EfficientNetB3 provides an ideal balance between predictive accuracy and computational effectiveness. Together, this integration is expected to yield higher diagnostic consistency and practical applicability in clinical settings. The key contributions of this work are summarized as follows,

- A novel DL approach is proposed by integrating the NSS with EfficientNetB3 architecture to improve OCT image representation and classification.
- OCT images are first transformed into the neutrosophic domain, where NSS highlights informative structures by suppressing noise, ambiguity and indeterminacy.
- A two‐phase training strategy is designed by combining frozen‐base training with steady fine‐tuning of deeper layers to improve feature learning.
- Extensive experiments on Retinal OCT dataset demonstrate that, NSS‐integrated EfficientNetB3 model outperforms other conventional DL models across multiple evaluation metrics, highlighting its potential for strong automated retinal disease classification.

The remainder of this paper is organized as follows. In Section 2, the materials and methods used in the paper are presented. The experimental results and discussion are presented in Section 3, while a comparative study with existing literature is presented in Section 4, followed by the Conclusion in the final Section.

## 2 MATERIALS AND METHODS

This section shows the methodological outline used for automated retinal disease classification using OCT images. The overall method integrates the image preprocessing, neutrosophic transformation, enhancement using the NSS combined with CLAHE and classification using the EfficientNetB3 model. Each component of the proposed approach is explained in detail in the following subsections.

### 2.1 Dataset description

This study uses a publicly available retinal OCT dataset containing of 24 000 images.^31^ The dataset is perfectly balanced in classes which containing 3000 images in each category. The data are arranged into predefined training, validation and test subsets consisting of 18 400, 2800 and 2800 images, respectively. In each category, there are 2300 images for training and 350 images each for validation and testing. The images were collected from multiple open‐access sources and were previously processed using standard procedures like cropping, padding and horizontal flipping to maintain consistent image dimensions. The balanced class splitting provides equal representation of all categories in each subset and provides a consistent and reproducible outline for model training, hyperparameter tuning and performance evaluation.

### 2.2 Proposed methodology

**FIGURE 1:**
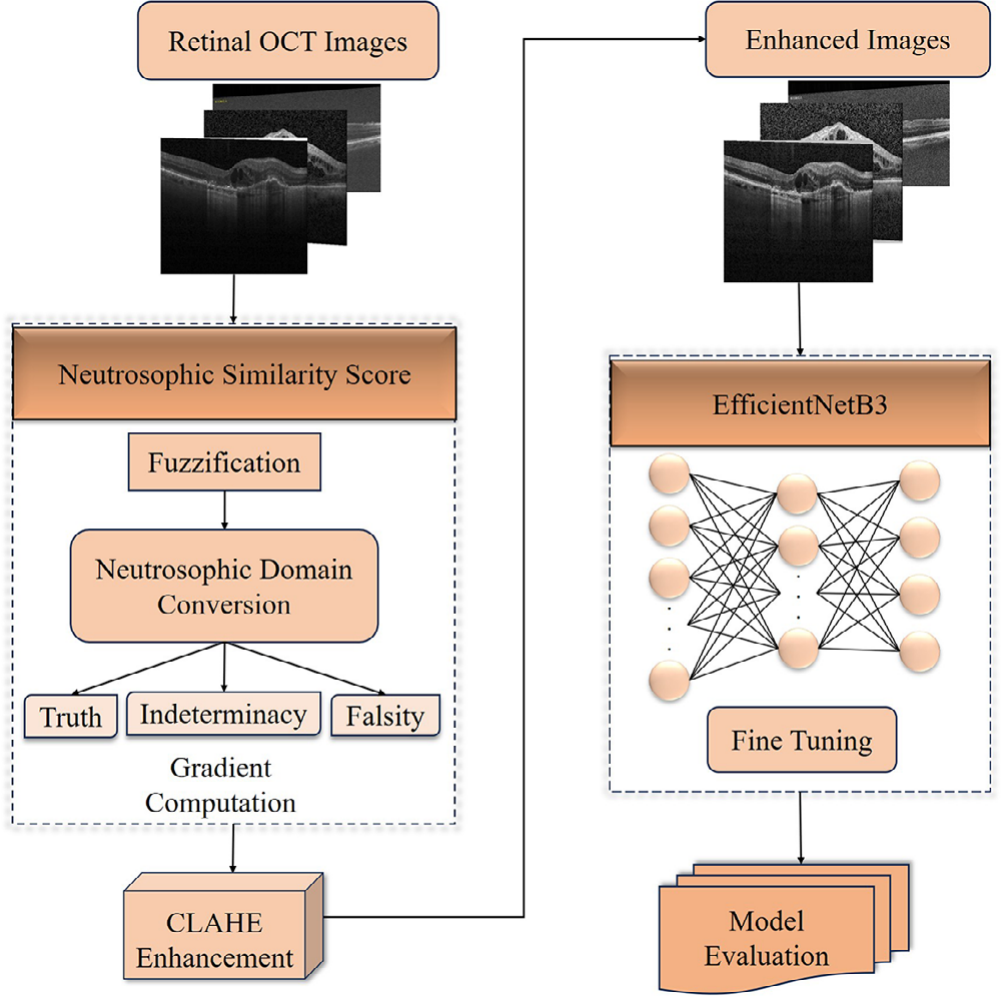
Work flow of the proposed image enhancement method.

The proposed methodology employs a structured workflow designed to enhance OCT images and improve the accuracy of retinal disease classification was illustrated in Figure 1. The process starts with preprocessing, where the input images are normalized and resized to ensure homogeneity across the dataset. Following this, the images were transformed into the neutrosophic domain to allow uncertainty and noise to be effectively modeled. The NSS is then applied to highlight diagnostically related regions while suppressing ambiguous areas. And then to further refine image quality, CLAHE is employed, which enhances local contrast and emphasizes refined structural features of the retina. The enhanced images are then processed by the EfficientNetB3 model and the DL architecture optimized for efficiency and accuracy, to perform multi‐class classification across eight retinal disease classes. This integrated pipeline combines advanced image enhancement with DL for enabling a strong feature extraction and reliable classification of OCT images.

### 2.3 Neutrosophic sets

Florentin Smarandache introduced the concept of NS in the 1990s^32^ and since then, neutrosophic logic was extensively adopted across various computer science domains, such as pattern recognition,^33^ image segmentation and image processing.^34^ A neutrosophic image is defined in the neutrosophic domain using three main components *T*, *I* and *F*. This formulation is based on neutrosophic set theory, which covers classical and fuzzy set outlines by explicitly modeling uncertainty and indeterminacy in data. In the Neutrosophic Domain, each pixel is characterized by three distinct values^35^

$$P_{ND}\left(x,y\right)=\left\{T\left(x,y\right),I\left(x,y\right),F\left(x,y\right)\right\}$$

(1) $$T_{C_{g}}\left(x,y\right)=\frac{g\left(x,y\right)-g_{min}}{g_{max}-g_{min}}$$

(2) $$I_{C_{g}}\left(x,y\right)=1-\frac{G_{d}\left(x,y\right)-{G_{d}}_{min}}{{G_{d}}_{max}-{G_{d}}_{min}}$$

(3) $$F_{C_{g}}\left(x,y\right)=1-T_{C_{g}}\left(x,y\right)$$

Where g(x,y) and $G_{d}\left(x,y\right)$ denote the intensity value and the gradient value at the pixel location (x,y) in the image.

### 2.4 Gradient computation using the sobel operator

To compute the indeterminacy component I(x,y), the image gradient is estimated using the sobel operator, which offers improved noise robustness and more accurate edge detection compared to the prewitt operator used in prior works.^33^ The sobel operator performs two‐dimensional discrete differentiation and emphasizes regions of high spatial frequency that correspond to significant intensity transitions. Let $I_{N}$ denote the normalized input image with pixel values scaled to the range [0, 1] and let ∗ represent the 2D discrete convolution operator. The horizontal $\left(S_{x}\right)$ and vertical $\left(S_{y}\right)$ Sobel kernels are defined as:

$$S_{x}=\left[\begin{array}{ccc} -1 & 0 & 1\\ -2 & 0 & 2\\ -1 & 0 & 1 \end{array}\right],S_{y}=\left[\begin{array}{ccc} -1 & -2 & -1\\ 0 & 0 & 0\\ 1 & 2 & 1 \end{array}\right]$$

This yields the gradient components, $G_{x}=I_{N}*S_{x},G_{y}=I_{N}*S_{y}$ which capture intensity variations along the x and y axes. The gradient magnitude is then computed as:

$$G_{d}\left(x,y\right)=\sqrt{{G_{x}}^{2}\left(x,y\right)+{G_{y}}^{2}\left(x,y\right)}$$

This gradient magnitude is then used in Equation (2) to compute the indeterminacy membership. The Sobel operator enhances fine structural details and suppresses noise, making it highly suitable for retinal OCT analysis.

### 2.5 Neutrosophic similarity score

The NSS algorithm^28^ was recently introduced and offers a novel approach to measuring the closeness of an object to the ideal reference. Its effectiveness in capturing and representing indeterminate characteristics has led to its widespread application across various domains. A neutrosophic set can be defined under different criteria, where the set of alternatives is denoted as $A=\{A_{1},A_{2},\text{…},A_{m}\}$ and the set of criteria as $C=\{C_{1},C_{2},\text{…},C_{J}\}$. The alternatives $A_{i}$ at $C_{j}$ criterion is denoted as $\left\{T_{C_{j}}\left(A_{i}\right),I_{C_{j}}\left(A_{i}\right),F_{C_{j}}\left(A_{i}\right)\right\}/A_{i}$, where $T_{C_{j}}\left(A_{i}\right),I_{C_{j}}\left(A_{i}\right)$ and $F_{C_{j}}\left(A_{i}\right)$ represent the *T*, *I* and *F* membership values of $A_{i}$ with respect to criterion $C_{j}$. The similarity score quantifies how closely two elements correspond within a neutrosophic set, especially in multi‐criteria decision‐making applications.

(4) $$\begin{array}{cc} & S_{C_{j}}\left(A_{m},A_{n}\right)=\\ & \frac{T_{C_{j}}\left(A_{m}\right)T_{C_{j}}\left(A_{n}\right)+I_{C_{j}}\left(A_{m}\right)I_{C_{j}}\left(A_{n}\right)+F_{C_{j}}\left(A_{m}\right)F_{C_{j}}\left(A_{n}\right)}{\sqrt{{T^{2}}_{C_{j}}\left(A_{m}\right)+{I^{2}}_{C_{j}}\left(A_{m}\right)+{F^{2}}_{C_{j}}\left(A_{m}\right)}\sqrt{{T^{2}}_{C_{j}}\left(A_{n}\right)+{I^{2}}_{C_{j}}\left(A_{n}\right)+{F^{2}}_{C_{j}}\left(A_{n}\right)}} \end{array}$$

The notion of an ideal element is employed to identify the most suitable alternative. The ideal alternative $A^{*}$ is defined as $\left\{{T^{*}}_{C_{j}}\left(A_{m}\right)+{I^{*}}_{C_{j}}\left(A_{m}\right)+{F^{*}}_{C_{j}}\left(A_{m}\right)\right\}/{A^{*}}_{i}$. The similarity between a given alternative and this ideal alternative is then computed as

(5) $$\begin{array}{cc} & S_{C_{j}}\left(A_{i},A^{*}\right)=\\ & \frac{T_{C_{j}}\left(A_{i}\right)T_{C_{j}}\left(A^{*}\right)+I_{C_{j}}\left(A_{i}\right)I_{C_{j}}\left(A^{*}\right)+F_{C_{j}}\left(A_{i}\right)F_{C_{j}}\left(A^{*}\right)}{\sqrt{{T^{2}}_{C_{j}}\left(A_{i}\right)+{I^{2}}_{C_{j}}\left(A_{i}\right)+{F^{2}}_{C_{j}}\left(A_{i}\right)}\sqrt{{T^{2}}_{C_{j}}\left(A^{*}\right)+{I^{2}}_{C_{j}}\left(A^{*}\right)+{F^{2}}_{C_{j}}\left(A^{*}\right)}} \end{array}$$

The ideal alternative $A^{*}$ has fixed membership values across all three criteria and is expressed as $\left\{{T^{*}}_{C_{j}}\left(A_{m}\right)+{I^{*}}_{C_{j}}\left(A_{m}\right)+{F^{*}}_{C_{j}}\left(A_{m}\right)\right\}/{A^{*}}_{i}=\left\{1,0,0\right\}/A^{*}$

Finally, to evaluate how closely an object corresponds to the ideal element under intensity conditions, a similarity score is calculated using the following formulation:

(6) $$\begin{array}{cc} & S_{C_{g}}\left(A_{i},A^{*}\right)=\\ & \frac{T_{C_{g}}\left(A_{i}\right)T_{C_{g}}\left(A^{*}\right)+I_{C_{g}}\left(A_{i}\right)I_{C_{g}}\left(A^{*}\right)+F_{C_{g}}\left(A_{i}\right)F_{C_{g}}\left(A^{*}\right)}{\sqrt{{T^{2}}_{C_{g}}\left(A_{i}\right)+{I^{2}}_{C_{g}}\left(A_{i}\right)+{F^{2}}_{C_{g}}\left(A_{i}\right)}\sqrt{{T^{2}}_{C_{g}}\left(A^{*}\right)+{I^{2}}_{C_{g}}\left(A^{*}\right)+{F^{2}}_{C_{g}}\left(A^{*}\right)}} \end{array}$$

### 2.6 CLAHE

**FIGURE 2:**
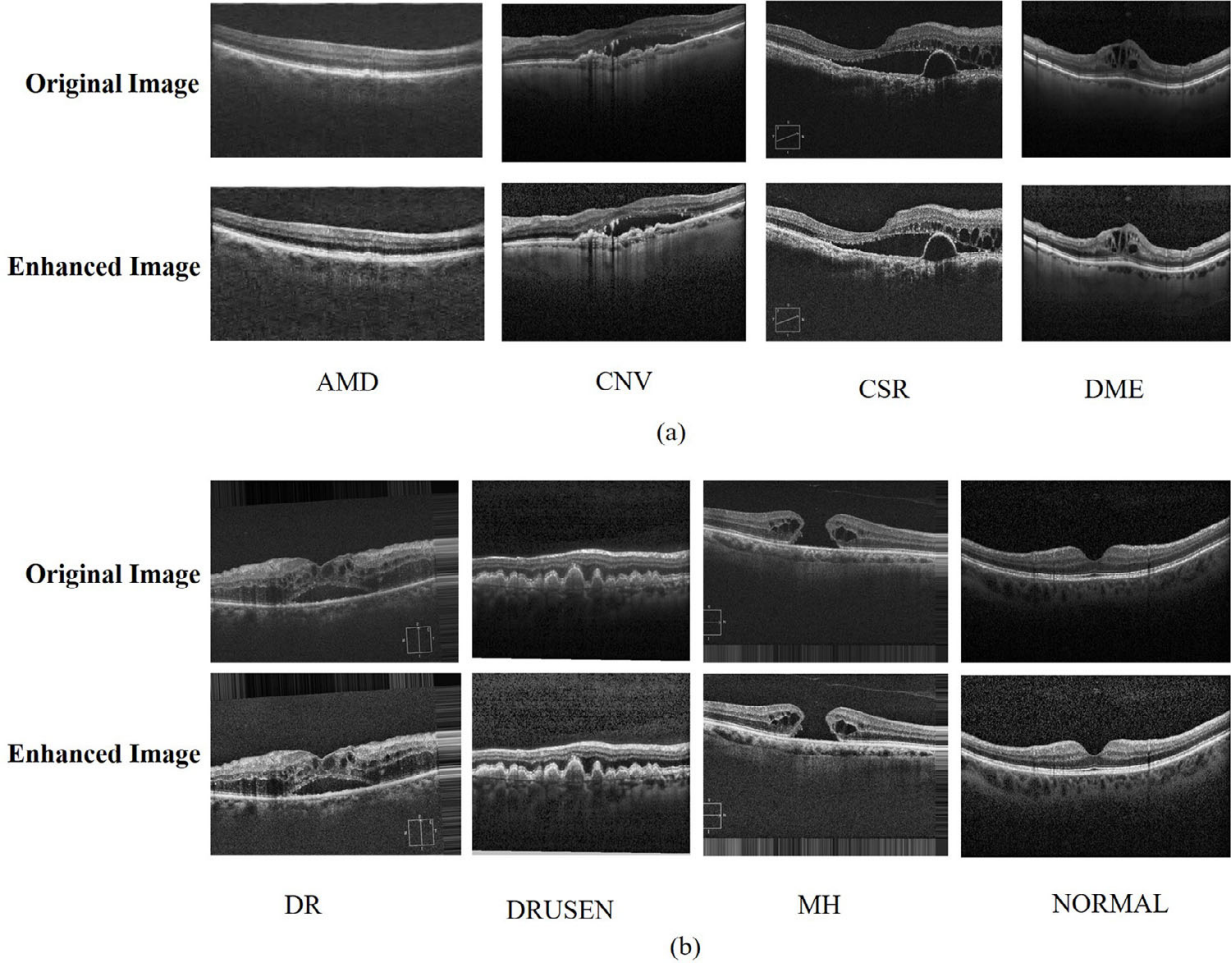
(a) NSS‐based enhancement results for AMD, CNV, CSR and DME OCT images; (b) NSS‐based enhancement results for DR, DRUSEN, MH and NORMAL OCT images.

To improve the local contrast of the image, NSS values are initially converted to 8‐bit format by scaling them by 255. Then, CLAHE^36^ is applied using a clip limit of 2.0 and an 8×8 tile grid to increase contrast by preventing unnecessary amplification of image details. The result is normalized by dividing it by 255 to bring values back to the [0,1] range for further processing. The CLAHE enhanced and normalized image shows better‐quality contrast along with stronger separation of the retinal layers and pathological structures. This preprocessing step was used to enhance the visibility of important image features, which supports more accurate classification. The complete enhancement process used for OCT images is described in Algorithm 1, which contains the transformation, analysis and contrast enhancement methods that are used to enhance image quality before classification. The visual effectiveness of this method is illustrated in Figure 2.

Algorithm 1 **NSS‐based enhancement of OCT images.**

**Table:** 

**Input** → Grayscale OCT image I(x,y)
**Output** → Enhanced image $\tilde{I}\left(x,y\right)$
*Parameters*:
ε ← 10^−^^8^ ▹ Small constant to avoid division by zero CLAHE clip ← 2.0 ▹ CLAHE clip limit CLAHE tile ← (8 × 8) ▹ CLAHE tile grid size α ← 255 ▹ 8‐bit scale
For each image I(x,y)∈ {Train, Val, Test}
**Step 1**: Normalization
$I_{min}\leftarrow \min\left(I\right)$ and $I_{max}\leftarrow \max\left(I\right)$
$I_{N}\left(x,y\right)\leftarrow \frac{I\left(x,y\right)-I_{min}}{I_{max}-I_{min}+\varepsilon }$
**Step 2**: Gradient estimation using Sobel operator
▹ $G_{x}\leftarrow I_{N}*S_{x},G_{y}\leftarrow I_{N}*S_{y}$
Compute gradient magnitude $G_{d}\left(x,y\right)\leftarrow \sqrt{{G_{x}}^{2}\left(x,y\right)+{G_{y}}^{2}\left(x,y\right)}$
**Step 3**: Neutrosophic components
$T_{C_{g}}\left(x,y\right)\leftarrow I_{N}\left(x,y\right)$
$I_{C_{g}}\left(x,y\right)\leftarrow 1-\frac{G_{d}\left(x,y\right)-{G_{d}}_{min}}{{G_{d}}_{max}-{G_{d}}_{min}}$
$F_{C_{g}}\left(x,y\right)\leftarrow 1-T_{C_{g}}\left(x,y\right)$
**Step 4**: NSS
$S_{C_{g}}\left(A_{i},A^{*}\right)=\frac{T_{C_{g}}\left(A_{i}\right)T_{C_{g}}\left(A^{*}\right)+I_{C_{g}}\left(A_{i}\right)I_{C_{g}}\left(A^{*}\right)+F_{C_{g}}\left(A_{i}\right)F_{C_{g}}\left(A^{*}\right)}{\sqrt{{T^{2}}_{C_{g}}\left(A_{i}\right)+{I^{2}}_{C_{g}}\left(A_{i}\right)+{F^{2}}_{C_{g}}\left(A_{i}\right)}\sqrt{{T^{2}}_{C_{g}}\left(A^{*}\right)+{I^{2}}_{C_{g}}\left(A^{*}\right)+{F^{2}}_{C_{g}}\left(A^{*}\right)}}$
**Step 5**: CLAHE
$NSS_{8bit}\leftarrow \left[\alpha .NSS(x,y)\right]$
$Apply\;CLAHE\leftarrow E_{norm}\left(x,y\right)$
**Step 6**: Defuzzification
$\tilde{I}\left(x,y\right)\leftarrow E_{norm}\left(x,y\right).\left(I_{max}-I_{min}\right)+I_{min}$

In order to increase the visual comparison presented in Figure 2 and to accurately evaluate the effectiveness of the proposed enhancement approach, quantitative image quality metrics was computed for representative samples from each retinal disease classes. peak signal‐to‐noise ratio (PSNR) and structural similarity index measure (SSIM) were employed to assess the protection of structural information and similarity between the original and the enhanced images. In addition, entropy was calculated to compute the changes in information content introduced by the enhancement procedure. This entropy reflects the richness of structural and textural information presented in the images. The computed results are summarized in Table 1 which provides quantitative evidence supporting the visual improvements attained through the proposed method.

**TABLE 1:** Quantitative results validating the effectiveness of the proposed enhancement approach across retinal OCT image classes.

Class	PSNR	SSIM	Entropy (original)	Entropy (enhanced)	Entropy improvement (%)
AMD	24.53 dB	0.8269	6.3862	6.6257	+3.8%
CNV	20.27 dB	0.6857	6.4183	6.7601	+5.3%
CSR	19.44 dB	0.7553	6.1010	6.9122	+13.3%
DME	21.41 dB	0.7188	6.4086	6.7254	+4.9%
DR	21.70 dB	0.7857	6.4847	7.0187	+8.2%
DRUSEN	19.40 dB	0.7211	6.7238	7.0891	+5.4%
MH	21.70 dB	0.7915	6.2679	6.9252	+10.5%
NORMAL	19.48 dB	0.6649	6.1746	6.5823	+6.6%

### 2.7 EfficientNetB3

**FIGURE 3:**
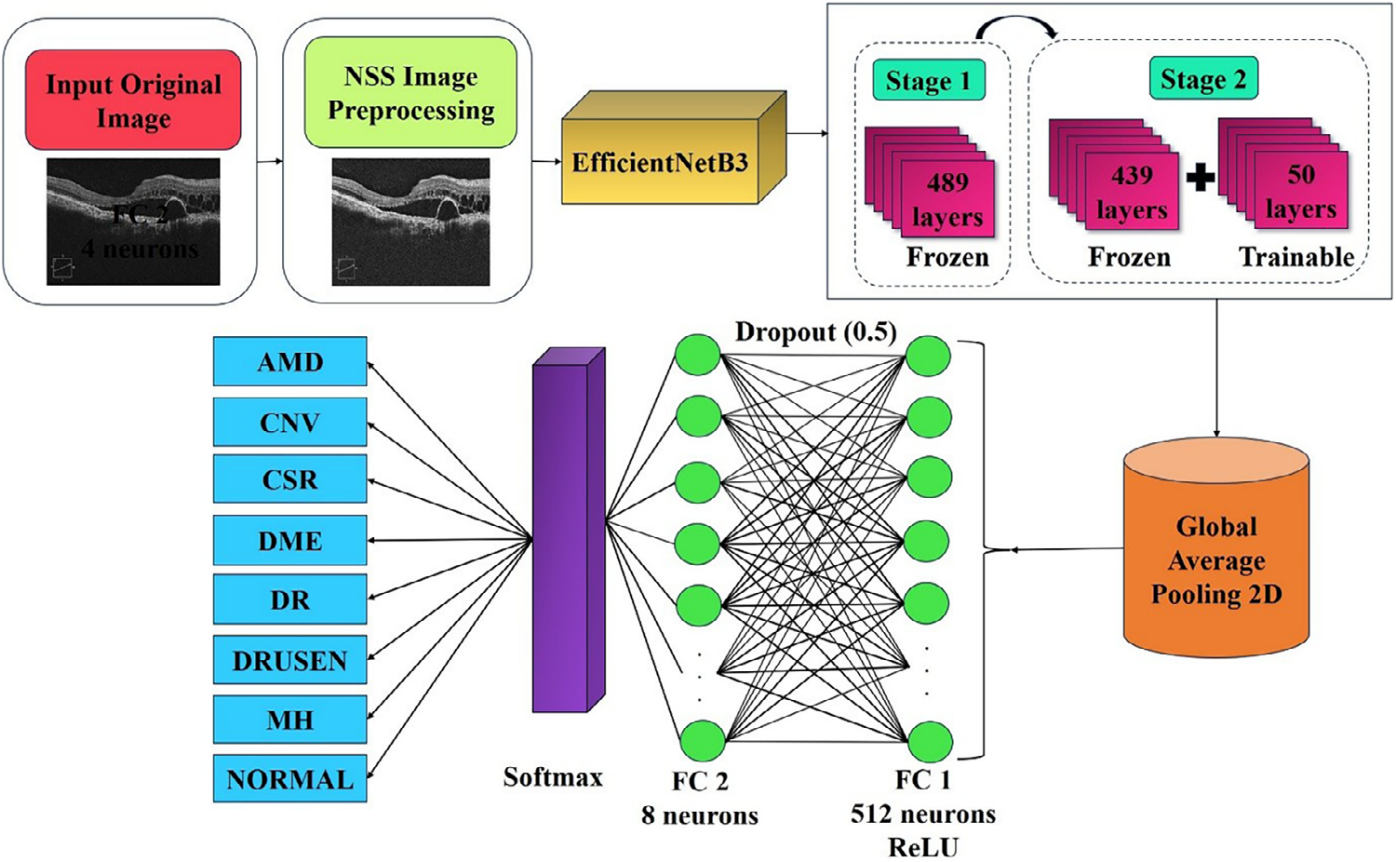
Workflow of the proposed NSS‐EfficientNetB3 framework illustrating preprocessing, dual‐stage fine‐tuning and multi‐class classification.

The EfficientNetB3 is part of EfficientNet series of CNNs, which was proposed by Tan and Le.^12^ Here, a compound scaling strategy is used to conjointly adjust network depth, width and input resolution which resulting in improved accuracy and computational efficiency. This model leverages mobile inverted bottleneck convolution (MBConv) blocks, that incorporates squeeze‐and‐excitation (SE) attention mechanisms and utilizes the swish activation function. It is designed to process input images of size 300×300 and EfficientNetB3 has around 12 million parameters that achieves a Top‐1 accuracy of approximately 81.6% on the ImageNet dataset. In spite of its high accuracy, it remains computationally effective on requiring only about 1.8 billion FLOPs making it a common choice for image classification tasks where the accuracy and efficiency are critical. The complete training strategy and architecture of the proposed NSS‐EfficientNetB3 outline was illustrated in Figure 3, and the detailed fine‐tuning process was summarized in Algorithm 2.

Algorithm 2 **EfficientNetB3 classification.**

**Table:** 

**Input** → Enhanced image $\tilde{I}\in R^{224\times 224\times 3}$
**Output** → Predicted label $\hat{y}\in R^{8}$
*1. Forward pass*
$f=M_{base}\left(\tilde{I}\right)$, $\hat{y}=Softmax\left(W.f+b\right)$
*2. Loss Categorical crossentropy*
$L=-\sum_{i=1}^{8}y_{i}\log\left(\hat{y_{i}}\right)$
*3. Training schedule*
***Stage 1*** Freeze $M_{base}$; Adam, $\eta =10^{-3}$, batch size = 32, epochs = 10 ***Stage 2*** Unfreeze last 50 layers; Adam, $\eta =10^{-4}$, batch size = 32, epochs = 20 ***Regularization*** Data augmentation + Dropout (0.5) ***Callbacks*** EarlyStopping *(restore best weights)*, ReduceLROnPlateau *(min lr =* $10^{-6}$)
4. *Evaluation* Report accuracy, precision, recall and F1 score on test set.

Following this architectural description, the EfficientNetB3 model was employed for multi‐class classification of retinal diseases using the Retinal OCT image dataset. The dataset comprised eight distinct classes, and all images were resized to 224×224 pixels to maintain uniformity. Training was performed using a batch size of 32 with the Adam optimizer and categorical cross‐entropy loss. To enhance generalization and minimize overfitting, comprehensive data augmentation techniques were applied, including random rotations, zooming, translations, brightness adjustments and horizontal flips. The model was initially trained for 10 epochs with its base layers frozen, followed by a second training phase in which the final 50 layers of the architecture were unfrozen and further trained for 20 epochs. This subsequent phase used a reduced learning rate of $1\times 10^{-4}$, enabling more refined deep feature extraction. Early stopping with best‐weight restoration and adaptive learning‐rate reduction were employed to stabilize training. The trained model obtained through this two‐stage fine‐tuning process was subsequently evaluated on the independent test set to assess classification performance, as discussed in the following section.

## 3 RESULTS AND DISCUSSION

**FIGURE 4:**
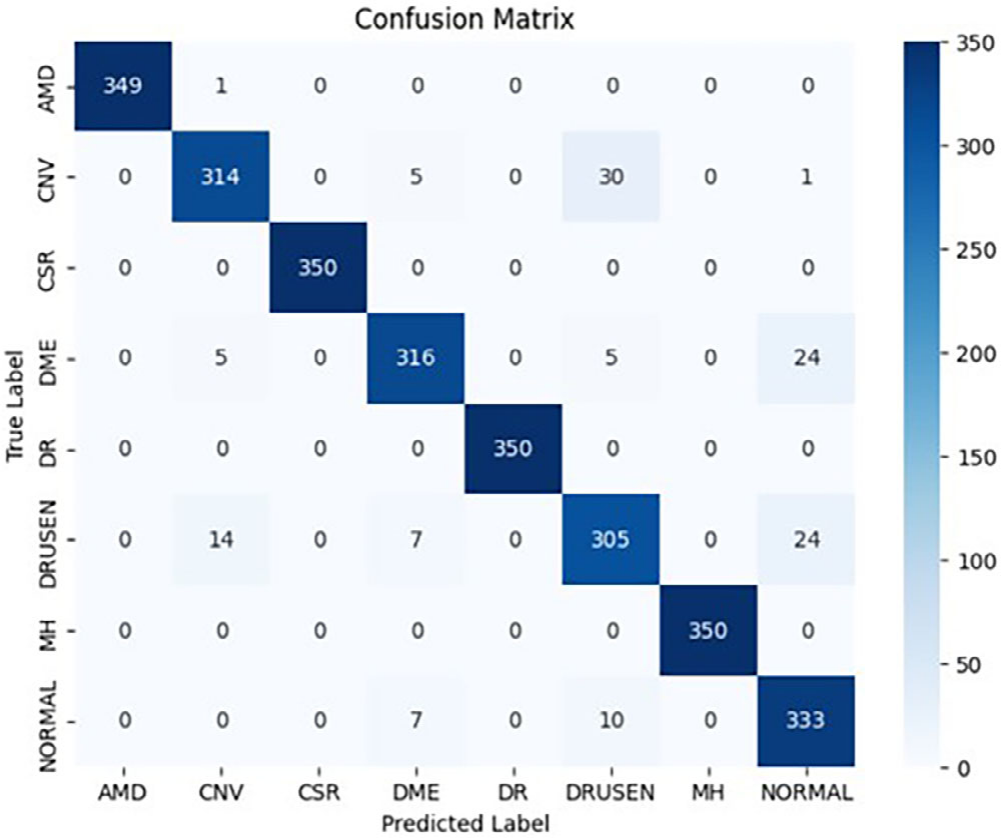
Confusion matrix showing the classification outcomes of the proposed model across eight retinal disease categories.

The experimental results obtained using the proposed NSS‐EfficientNetB3 approach are presented and analyzed in this section to evaluate its effectiveness in multi‐class retinal OCT image classification. The following discussion focuses on numerical performance metrics and class‐wise prediction behavior of the model. This training pipeline was implemented on a neutrosophic‐enhanced version of the retinal OCT dataset, where the images are preprocessed using a neutrosophic transformation method which was designed to address uncertainty, indeterminacy and noise. The model was trained on the enhanced dataset, which delivered improved classification results, achieving a test accuracy of 95.25%, with precision, recall and F1 score of 95.33%, 95.25% and 95.26%, respectively. These results confirm the effectiveness of the proposed approach in carrying a strong and well‐generalized model that is capable of accurately distinguishing between all eight retinal disease classes. The confusion matrix, shown in Figure 4, illustrates the classification results across all the classes which highlights near‐perfect recognition for most classes such as AMD, CSR, DME and MH, by demonstrating a strong performance in more challenging cases like CNV and DRUSEN, where only minimal misclassifications was occurred.

**FIGURE 5:**
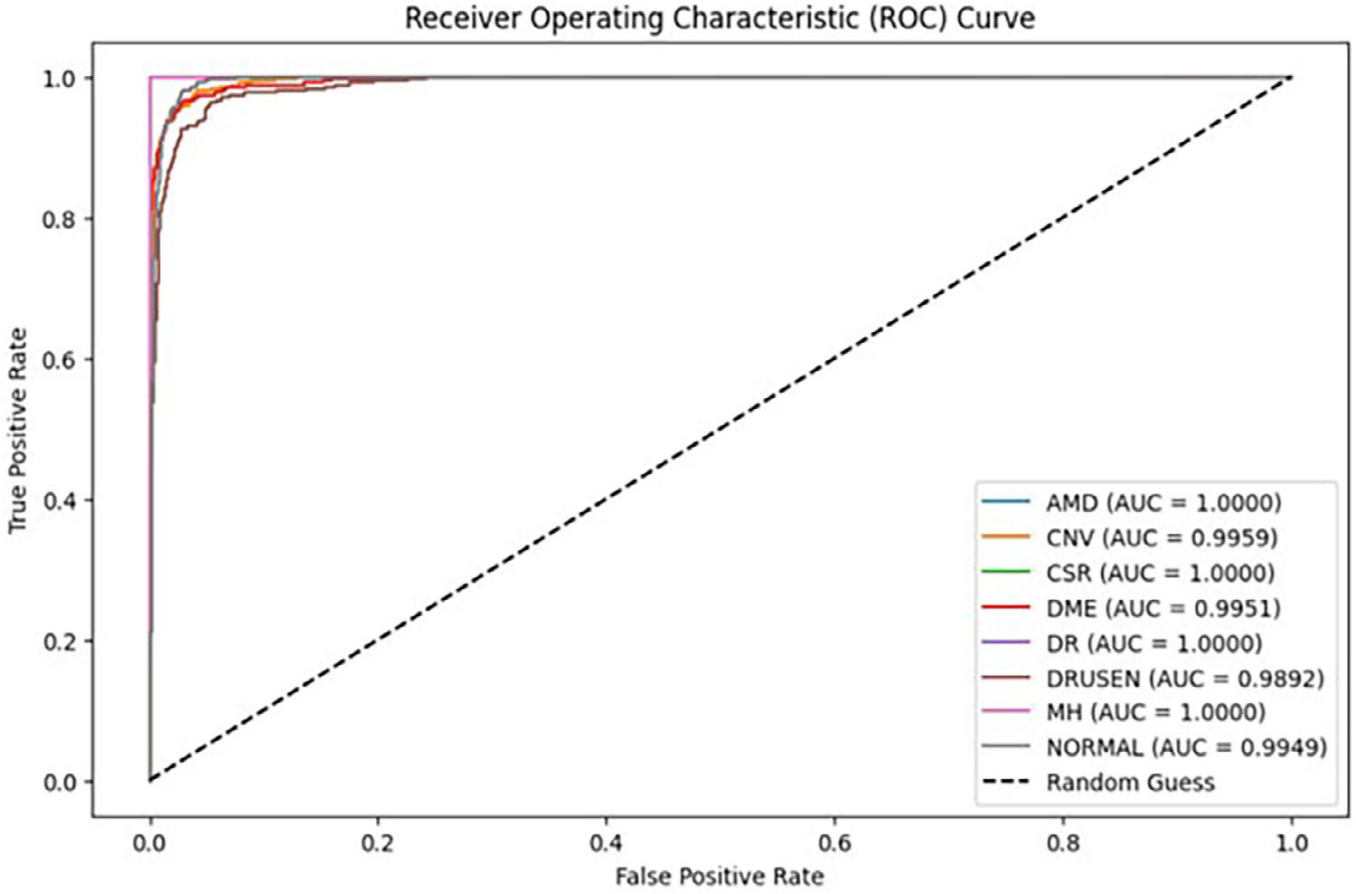
ROC curves illustrating the discriminative performance of the proposed model for all classes.

The Receiver Operating Characteristic (ROC) curve, which is shown in Figure 5, further validates the discriminative power of the model. The area under the curve values is constantly above 0.99 for all classes, with several categories (AMD, CSR, DME and MH) achieving a perfect AUC of 1.0, that highlighting the model's reliability and high sensitivity in distinguishing refined variations among the retinal diseases. Together with these results, the proposed approach provides strong evidence of its potential for clinical application in automated retinal disease diagnosis.

The comparative evaluation process began with an evaluation of the proposed NSS‐EfficientNetB3 approach against broadly used DL architectures on the same dataset. As shown in Table 2, the baseline EfficientNetB3 model trained without any enhancement was observed to achieve an accuracy of 87.11%. However, the proposed model was observed to achieve a significantly high accuracy of 95.25%. Similar improvements were consistently observed across precision, recall and F1‐score, representing the contribution of neutrosophic preprocessing and the two‐phase training strategy. The other architectures, such as MobileNetV2, DenseNet121, InceptionV3 and Xception, also yielded comparatively lower results which confirming the robustness of the proposed pipeline.

**TABLE 2:** Comparison of proposed work with other DL models.

Model	Accuracy	Precision	Recall	F1 score
MobileNetV2	0.8382	0.8434	0.8382	0.8384
VGG16	0.7425	0.7516	0.7425	0.7411
VGG19	0.7425	0.7516	0.7425	0.7411
DenseNet121	0.8564	0.8630	0.8564	0.8562
InceptionV3	0.8479	0.8613	0.8479	0.8475
Xception	0.8286	0.8297	0.8286	0.88278
EfficientNetB3	0.8711	0.8737	0.8711	0.8715
NSS‐EfficientNetB3	**0.9525**	**0.9533**	**0.9525**	**0.9526**

**FIGURE 6:**
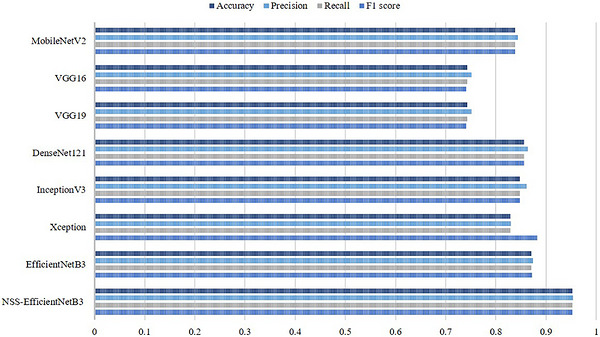
Graphical representation of NSS‐EfficientNetB3 performance compared with other DL models.

In addition to that, the incorporation of neutrosophic preprocessing not only improved the structural description of the retinal features but also improved the consistency of the model predictions, as seen in the higher evaluation metrics. The effectiveness of the proposed approach can be further seen in Figure 6, which provides a graphical representation comparison between the EfficientNetB3 model and other DL architectures. From this comparison, it clearly demonstrates that the proposed approach consistently outperforms the baseline models across all the major evaluation metrics. These results confirm the effectiveness of combining advanced image enhancement methods with a well‐structured training pipeline for improved medical image classification.

To further investigate the contribution of each component in the proposed framework, an ablation study was carried out by conducting various preprocessing configurations and as well as training approaches. The results of this study are presented in Table 3. The goal was to measure the impact of NSS‐based image enhancement, CLAHE‐based contrast normalization as well as fine‐tuning of EfficientNetB3 in classification.

**TABLE 3:** Ablation study of the proposed NSS‐EfficientNetB3 framework.

Model Configuration	Accuracy (%)	Precision (%)	Recall (%)	F1‐score (%)
Raw OCT images + EfficientNetB3	87.11	87.37	87.11	87.15
NSS + EfficientNetB3	88.68	88.81	88.68	88.67
CLAHE + EfficientNetB3	89.25	89.60	89.25	89.23
NSS + EfficientNetB3 with fine‐tuning	94.61	94.81	94.61	94.62
CLAHE + EfficientNetB3 with fine‐tuning	94.50	94.72	94.50	94.52
**Proposed: NSS + CLAHE + EfficientNetB3 with fine‐tuning**	**95.25**	**95.33**	**95.25**	**95.26**

The ablation studies have demonstrated that the NSS and CLAHE preprocessing techniques individually enhance classification performance over the raw baseline and with CLAHE, it provides a slightly larger gain in this configuration (+2.08% in F1‐score). Then fine‐tuning the last 50 layers of EfficientNetB3 yields significant improvements across all variants (approximately 5 to 7% absolute in F1‐score), demonstrating the critical role of domain‐specific adaptation for retinal OCT images. The proposed approach combining the NSS and CLAHE preprocessing with fine‐tuning has achieved the highest performance (95.26% F1‐score) and confirming that the two enhancement techniques provide complementary benefits. The NSS supports structure‐preserving enhancement while CLAHE handles local contrast adaptation which is then resulting in the most effective classification framework.

## 4 COMPARATIVE ANALYSIS

To better assess the proposed approach from the existing context of research, a comparative analysis of the results obtained in the relevant literature was performed. For the eight‐class classification set‐up, the cited studies were employed the same publicly available retinal OCT dataset, so that it enabling a meaningful reference comparison. However, the differences in preprocessing approaches, data augmentation, training procedures and experimental settings across studies may impact described performance. Therefore, the comparison was presented in Table 4 should have been construed as an indicative benchmark rather than a strictly controlled experimental evaluation. Despite of these variations, the proposed NSS‐EfficientNetB3 approach demonstrates competitive and consistent performance across all evaluation metrics.

**TABLE 4:** Comparative evaluation of the proposed NSS‐EfficientNetB3 framework against existing OCT image classification techniques for four‐class and eight‐class categories.

Author	Method	Class	Accuracy
Yoo et al.^37^	GAN	4	0.912
Sharon et al.^38^	Ensemble Learning	4	0.9289
Karthik et al.^39^	ResNet34	8	0.924
Karthik et al.^39^	ResNet50	8	0.903
Karthik et al.^39^	ResNet101	8	0.861
He et al.^40^	ViT	8	0.8896
He et al.^40^	Swin‐Transformer	8	0.9454
**Proposed**	**NSS‐EfficientNetB3**	**8**	**0.9525**

Overall, the results show that the proposed methodology not only enhances the structural representation of the retinal features but also provides improved consistency and reliability of the classification results. By achieving better performance than both the baseline models and the existing literature, the framework shows its potential as a practical tool for automated retinal disease diagnosis from OCT scans. These findings support the importance of advanced image enhancement techniques in addressing the limitations of OCT imaging and highlight the ability of neutrosophic‐based preprocessing for future clinical decision‐support systems.

## 5 CONCLUSION

The proposed study has shown the efficiency of neutrosophic image enhancement as a preprocessing technique for classifying retinal disease using OCT scans. By transforming the images into neutrosophic domain, the proposed approach enhances the meaningful structural details by reducing noise and uncertainty and thus providing a more informative representation for DL models. When integrated with the EfficientNetB3 architecture, the proposed framework achieved significant improvements across all the major evaluation metrics when compared with models trained on original and unprocessed datasets. The proposed framework is also found to enhance the consistency of learning as well as improve the generalization performance of learning in an eight‐class classification problem. This further emphasizes the need to effectively preprocess images in medical imaging problems. Overall, it is clear that the integration of neutrosophic enhancement with an EfficientNetB3‐based DL architecture presents a reliable approach for automated retinal disease classification and establishes a strong foundation for developing accurate and practical computer‐aided diagnostic systems. Furthermore, by connecting image enhancement with improved algorithmic performance, the proposed approach highlights a capable direction for advancing automated retinal disease analysis and supports future research toward reliable, scalable and clinically meaningful diagnostic solutions.

## AUTHOR CONTRIBUTIONS

**Devi M**: Conceptualization; methodology; software; formal analysis; investigation; data curation; validation; visualization; roles/writing—original draft; writing—review & editing. **G. Hannah Grace**: Supervision; resources; writing—review & editing.

## CONFLICT OF INTEREST STATEMENT

The authors declare no conflicts of interest.

## Data Availability

The dataset used in this study is publicly available from the Kaggle Retinal OCT dataset. The data can be accessed at: https://www.kaggle.com/datasets/obulisainaren/retinal‐oct‐c8/data
